# The ontology of medically related social entities: recent developments

**DOI:** 10.1186/s13326-016-0087-8

**Published:** 2016-07-12

**Authors:** Amanda Hicks, Josh Hanna, Daniel Welch, Mathias Brochhausen, William R. Hogan

**Affiliations:** Department of Health Outcomes and Policy, University of Florida, Gainesville, FL USA; Department of Biomedical Informatics, University of Arkansas for Medical Science, Little Rock, AR USA

**Keywords:** Ontology, OMRSE, Social entities, Social roles, Organizations

## Abstract

**Background:**

The Ontology of Medically Related Social Entities (OMRSE) was initially developed in 2011 to provide a framework for modeling demographic data in Resource Description Framework/Web Ontology Language. It is built upon the Basic Formal Ontology and conforms to Open Biomedical Ontologies Foundry’s best practices.

**Description:**

We report recent development of OMRSE which includes representations of organizations, roles, facilities, demographic data, enrollment in insurance plans, and data about socio-economic indicators.

**Conclusions:**

OMRSE’s coverage has been expanding in recent years to include a wide variety of classes and has been useful in several biomedical applications.

## Background

The Ontology of Medically Related Social Entities (OMRSE) [1] is a realist representation of medically related social entities. We initially developed OMRSE to cover demographics data and common roles of people in healthcare encounters for reuse in the context of the OBO Foundry [1]. We created a framework for defining gender roles, legal roles, healthcare provider roles, healthcare organization roles, and patient roles in Web Ontology Language (OWL), one of the accepted languages for the OBO Foundry and a standard for the Semantic Web. We have since developed this ontology by adding more specific classes and creating frameworks for additional topics to facilitate uses arising out of projects related to epidemic modeling, the organizational structure of trauma systems, and common health care data models. OMRSE is a middle level ontology in the sense described in [2]. It is designed to bridge the gap between the upper ontology, Basic Formal Ontology (BFO), and more specific domain ontologies as well as provide classes for reuse in application ontologies. While we acknowledge that the demarcation between middle and domain level ontologies is not crisp [2], OMRSE contains mid-level classes such as employee role, smoker role, and party to a marriage contract that span multiple sub-domains and can be reused in both more specific domain ontologies as well as application ontologies.

## Applications

OMRSE classes are reused in several application ontologies. It is available to the wider biomedical community through OntoBee [3] and NCBO Bioportal [4].

The Apollo [5] and MIDAS projects reuse OMRSE classes in Apollo-SV to produce synthetic ecosystems for agent based epidemic modeling. The CAFÉ Project reuses OMRSE classes in the Ontology of Organizational Structures of Trauma centers and Trauma systems (OOSTT) (http://purl.obolibrary.org/obo/oostt.owl). OOSTT is an OWL representation of organizational structures (organizations, committees, roles, etc.) specific to trauma centers or trauma systems. It is used to compare the organizational structure of trauma centers and trauma systems. OMRSE classes are also being used to create a semantic representation of the PCORnet Common Data Model (CDM).

## Construction and content

In keeping with the OBO Foundry principles [1], OMRSE reuses classes from other ontologies including the Basic Formal Ontology (BFO) [6], NCBI Taxonomy [7], the Information Artifact Ontology [8], and the Document Acts Ontology (D-acts) [9]. We searched OntoBee or Bioportal for existing classes in BFO-based ontologies before adding new ones to OMRSE. We participate in discussion about BFO and other OBO ontologies to ensure compatibility with other OBO/BFO ontologies. The additions that we report in this paper have been developed with investigators on these projects to ensure accurate and useful semantics of new classes. The recent developments in OMRSE can be classified into eight content areas described in the subsections below. In addition to introducing new content, we have also updated OMRSE to be compatible with the Basic Formal Ontology 2.0 (BFO2).

### Organizations and the roles they create

Some roles, such as employee and student roles, exist only in relation to organizations. To represent the relationships between these roles and organizations we leveraged the work in D-Acts, an OWL ontology built according to the OBO Foundry principles and using BFO. D-acts is based on works by Reinach [10] and Smith [11] explaining how social acts create new entities. The ontology represents social acts (such as signing a contract or enrolling as a student), the socio-legal entities those social acts create (such as rights and obligations or a student role), and the object properties relating these kinds of entities [10].

### Demographic data

The major developments for representing demographic data consist in modeling social identities (race and ethnicity) and marital status. Social identities differ from other demographic data since the referent of identities is ontologically unclear. For considerations of space and clarity, we reserve a discussion of social identities for a future manuscript. At present details can be found in [12] and https://www.youtube.com/watch?v=-pcQUNWtnVk.

#### Marital status

Many coding schemes have several values for marital status (HL7 has nine), but we model marital status as binary. In clinical settings marital status is recorded to document whether a patient has a spouse who can make decisions on his or her behalf; either they have a spouse to make decisions on their behalf or they do not. We currently have no use case for knowing a person has never been married nor for capturing what process resulted in the end of a marriage (i.e., death of the spouse or a divorce).

### Typology of trauma patients

We worked with a team of trauma experts who identified and reviewed definitions for trauma patients for the The CAFÉ project (1R01 GM111324). Current classes include injured patient role, burn patient role, and trauma patient role. A typology of burn patients was also defined. We plan to add these classes once there are suitable classes for types of burns (e.g., thermal vs. chemical burns) in another OBO ontology.

### Health care facilities

We developed a typology of twelve types of health care facilities that are referred to in the PCORnet CDM’s discharge status field in the encounters Table [13] (e.g., hospital facility, urgent care facility, nursing home facility). We distinguish the types of facilities based on their functions

### Health care provider roles

We have distinguished health care provider roles along the lines of what kind of entity can bear that role. Accordingly, we have health care provider organization role and subclasses that inhere in organizations. These subclasses are, hospital role, integrated delivery network, and physician practice. We also have health care provider role as a subclass of human health care role. These include nurse role, physiatrist role, physician role, and US physician role.

### Smoking statuses

OMRSE captures smoking status using smoker roles. Smoker role is defined as “a role that inheres in an human being and is realized by habitually smoking tobacco products.” The subclasses heavy smoker role and light smoker role are defined in terms of number of cigarettes habitually smoked per day. Further distinctions based on smoke exposure and source can be added as applications require them.

### Enrollment in an insurance plan

Modeling enrollment in an insurance plan requires modeling three different types of entities: (1) insurance policies, (2) the roles involved in an insurance policy, and (3) enrollment dates.

#### Insurance policies

Insurance policies come into existence through document acts. In technical terms, they are the specified output of document acts. The document acts involve two parties (1) a group of persons (the insured parties) and (2) the organization that issues the plan. The organization and the primary insured persons on the policy are parties to a legal agreement (an insurance policy).

#### Roles

There are two types of roles introduced to model insurance policies: those that inhere in the insurance company and those that inhere in the insured. More details about how these roles are modeled are available at http://ncor.buffalo.edu/2016/Hicks.pptx.

### U.S. Census households and housing units

Households and housing units are pivotal for representing U.S. Census data and epidemic modeling. OMRSE represents the distinction between households, which are collections of people, and housing units and asserts that housing units are individuated by their residence functions.

### Socio-economic data

Although we do not directly represent socio-economic status, with the exception of employee role and insurance enrollment information, we do represent data that are about socio-economic status. These terms are included to facilitate the annotation of data sets that contain information about employment status, care plans, income, and other socio-economic indicators.

### BFO 2.0 Conversion

The only modification to OMRSE that was required to complete this conversion was to import version 2015-10-07 of the Relation Ontology [14].

## Utility and discussion

### Validation

We have generated competency questions to validate the following representations: (1) employee roles, student roles, and household data, and (2) health care organizations and the typology of patients. Competency questions and queries for (1) are freely available at www.github.com/ufbmi/socid and http://tinyurl.com/syneco-queries respectively. Competency questions have been developed for OOST, but OOST is still under development and has not yet been validated. Table 1 has sample competency questions.

**Table 1:** Sample competency questions

Topic	Ontology	Question
Demographics	PCORowl	Who has identified as Asian
		according to both OMB and
		PCORnet CDM guidelines?
Marital status/households	MIDAS	Who are the married
		householders according to the
		U.S. Census?
Roles and organization	CAFÉ	How many anesthesiologists
		does institution x have on staff?

### Limitations

The typologies that we mention here are not exhaustive. For instance, the typologies of health care provider roles and patient roles are relatively sparse compared to the myriad of roles that a provider or patient might bear. This is the result of our use case driven approach. We maintain an active mailing list and issue tracker to manage requests to the ontology.

Stating that an individual person is ‘single’, i.e. lacks a party to a marriage contract role, in OWL is challenging but also necessary to support modeling demographic data in a manner that is compliant with OBO Foundry principles. Two approaches are (1) using negative object property axioms and (2) defining a class ‘single person’ as a person who is not the bearer of a party to a marriage contract role. The former is often not supported by common reasoners and the latter would lead to a proliferation of “absence” classes for every case where some individual lacks a relationship to some class of entities. The analogous problem is still outstanding for non-smoker, for example. A detailed description of this problem goes beyond the scope of reporting updates, but we will address this in future work and are currently investigating the pros and cons of these approaches, as well as attempting to come up with additional approaches.

## Conclusion

OMRSE is an ontology designated for representing medically related social entities in a manner that is consistent with BFO and OBO Foundry ontologies. Its coverage has been expanding in recent years to include a wide variety of classes.. and has been useful in several biomedical applications.

## Availability and requirements

OMRSE is free and open to all users (https://github.com/ufbmi/OMRSE). There is a Google Group for discussing the project at http://groups.google.com/group/omrse-discuss.
